# Stray light characterization with ultrafast time-of-flight imaging

**DOI:** 10.1038/s41598-021-89324-y

**Published:** 2021-05-12

**Authors:** L. Clermont, W. Uhring, M. Georges

**Affiliations:** 1 Centre Spatial de Liège, STAR Institute, Université de Liège, Avenue du Pré-Aily, 4031 Liège, Belgium; 2 ICube Research Institute, University of Strasbourg and CNRS, 23 rue du Loess, 67037 Strasbourg Cedex, France

**Keywords:** Techniques and instrumentation, Applied optics, Optical techniques

## Abstract

Understanding stray light (SL) is a crucial aspect in the development of high-end optical instruments, for instance space telescopes. As it drives image quality, SL must be controlled by design and characterized experimentally. However, conventional SL characterization methods are limited as they do not provide information on its origins. The problem is complex due to the diversity of light interaction processes with surfaces, creating various SL contributors. Therefore, when SL level is higher than expected, it can be difficult to determine how to improve the system. We demonstrate a new approach, ultrafast time-of-flight SL characterization, where a pulsed laser source and a streak camera are used to record individually SL contributors which travel with a specific optical path length. Furthermore, the optical path length offers a means of identification to determine its origin. We demonstrate this method in an imaging system, measuring and identifying individual ghosts and scattering components. We then show how it can be used to reverse-engineer the instrument SL origins.

## Introduction

**Figure 1:**
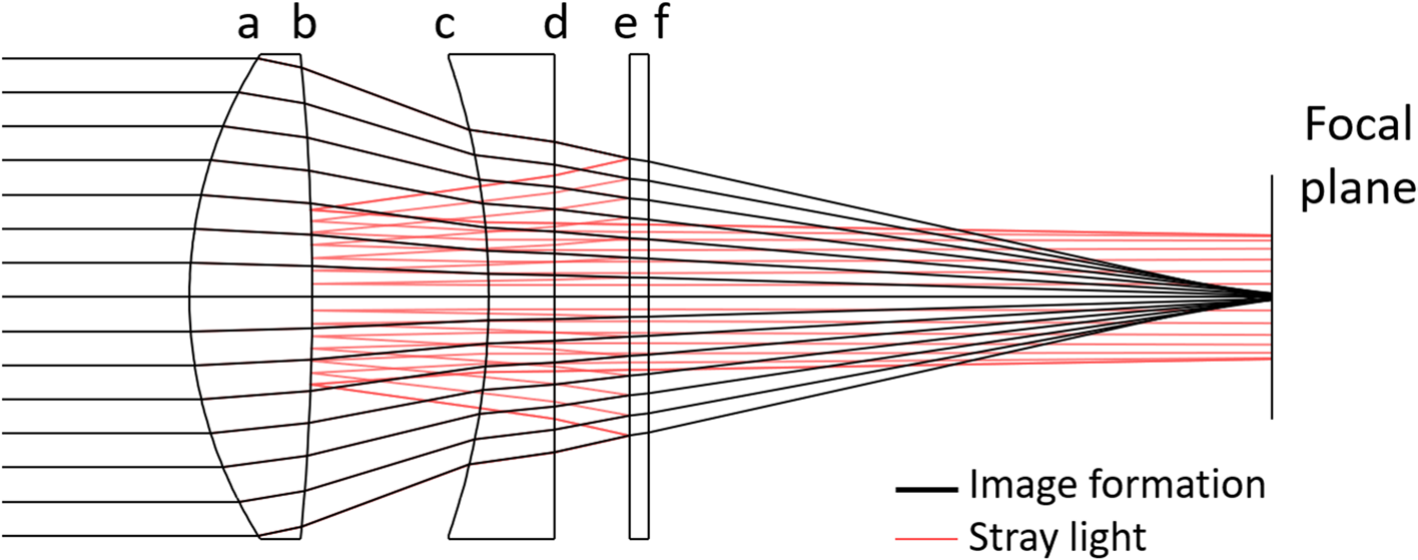
Sketch of the optical system under test, composed of two lenses and an optical window. An object at infinity is imaged as a point at the focal plane. Paths such as ghost reflections between surfaces *e* and *b* create SL at the focal plane. The elements are mechanically supported by spacers inside a tube.

The image formed by an optical system is degraded when stray light (SL) reaches the detector^1^. SL can appear through partial reflection between the lens interfaces (ghosts) (Fig. 1), or from scattering on the housing or due to the roughness of optical elements. Unwanted features in the image and resolution loss are direct consequences. SL can potentially appear in any kind of optical instruments, from personal cameras to microscopes^2,3^ among others. However, the criticality of SL depends on the application. In the particular case of space optical instruments, SL is a key aspect as it can make or break a mission. For example, SL affects the radiometric accuracy of Earth observation instruments. It can also jeopardize the detection of faint objects such as exoplanets in the case of coronagraphs. The worst situation is if a SL issue is only discovered once the instrument is already in space, as it was the case for example in the GAIA mission^4^.

For these reasons, SL mitigation is inherent of every steps in instruments development. Ray-tracing allows prediction of SL paths, which are then suppressed or attenuated with baffles or coatings^1,5,6^. Afterwards, SL is characterized experimentally to verify the performance. This is done by illuminating the system with a point-like source, returning an image of the different SL components superposed with the nominal image. Such process does not allow to decompose and retrieve the origin of the individual SL components. Therefore, if the measured SL is higher than expected, improvement actions are limited^5^. The inability to decompose and identify SL contributors is a major drawback for engineers who can only guess SL origins and use a trial and error approach for improving a system. This is highly time consuming and sometimes no solution is found.

In this work, we demonstrate how ultrafast time-of-flight imaging (ToF) offers a new paradigm in SL characterization. As SL components have different optical path lengths (OPL), illumination with a pulsed laser beam and detection with an ultrafast camera allows to characterize them separately. Furthermore, the OPL offers a means of identification to determine their origin. With these capabilities, major limitations of current methods will be overcome, paving the way to new possibilities in the development of space optical instruments. In the demanding case of refractive assemblies, SL components with typical millimeter path length differences should be made observable with temporal resolution of a few picoseconds. High spatial resolution is also desired for unequivocal characterization of SL.

Recent improvements of 2D fast sensors based on single-photon avalanche diodes (SPADs) make them an interesting candidate technology for ultrafast ToF imaging, with increasing numbers of pixels^7,8^. Latest works with SPADs also involve occluded scene reconstruction^9–11^. Nevertheless, the reported temporal resolution (a few tens of picoseconds) remains too small for our application. An alternative is the use of streak cameras which allow visualizing time arrival of photons from the scene along the unidimensional direction of the streak tube entrance slit^12^. Various studies showed their use as a framing camera to allow single shot direct imaging of 3D scenes with a few ps of temporal resolution but the spatial resolution remains limited^13–17^. In our application, the scene is static and repeated pulsed illumination with a scanning of the slit allows to reconstruct images with high spatial and temporal resolutions. In this frame, considerable breakthroughs in 3D scene reconstructions by ToF imaging were made by MIT^18–23^. Before reaching the streak tube, a pulsed laser beam travelling through a scene interacts with objects through a variety of processes: specular and diffuse reflection (sometimes multiple times), subsurface interactions, or diffraction. Suitable processing allows applications such as observing occluded objects^18,19^, observing light travelling through complex scenes^20,21^, retrieving three-dimensional reflectance function of surfaces on very-wide angles^22^, or more recently multi-zoom and multi-spectral imaging^23^. The principle of ToF imaging with streak cameras can be understood as tagging temporal arrival of photons in a spatial dimension. Another possibility is to use white light pulses to illuminate the scene and then to tag arrival of photons spectrally, as demonstrated by Goda et al^24^ and later by Nakagawa et al^25^. This method is not relevant for our application since SL is spectrally dependent and its characterization is envisaged at a specific wavelength. Finally, a method called light-in-flight holography, popularized by Abramson^26,27^ allows tagging temporal photons through spatial multiplexing in holographic plates^28,29^, or more recently via digital hologram recording^30^, making use of low-coherence light pulses. Despite the interesting spatial and temporal resolution (up to ~ 100 fs^30^), holography seems unsuitable for recording multiple SL components over a large dynamic range.

Therefore, the method that appeared the most suited for our application was that of ultrafast ToF imaging based on streak cameras^20,21^. In this paper, ultrafast ToF imaging at ps-scale for retrieving unequivocally and characterizing SL components in an optical system, whether they are generated by ghost reflections or scattering. The different contributors are isolated and reverse engineering of the instrument properties is achieved.

## Results

### Experimental setup

**Figure 2:**
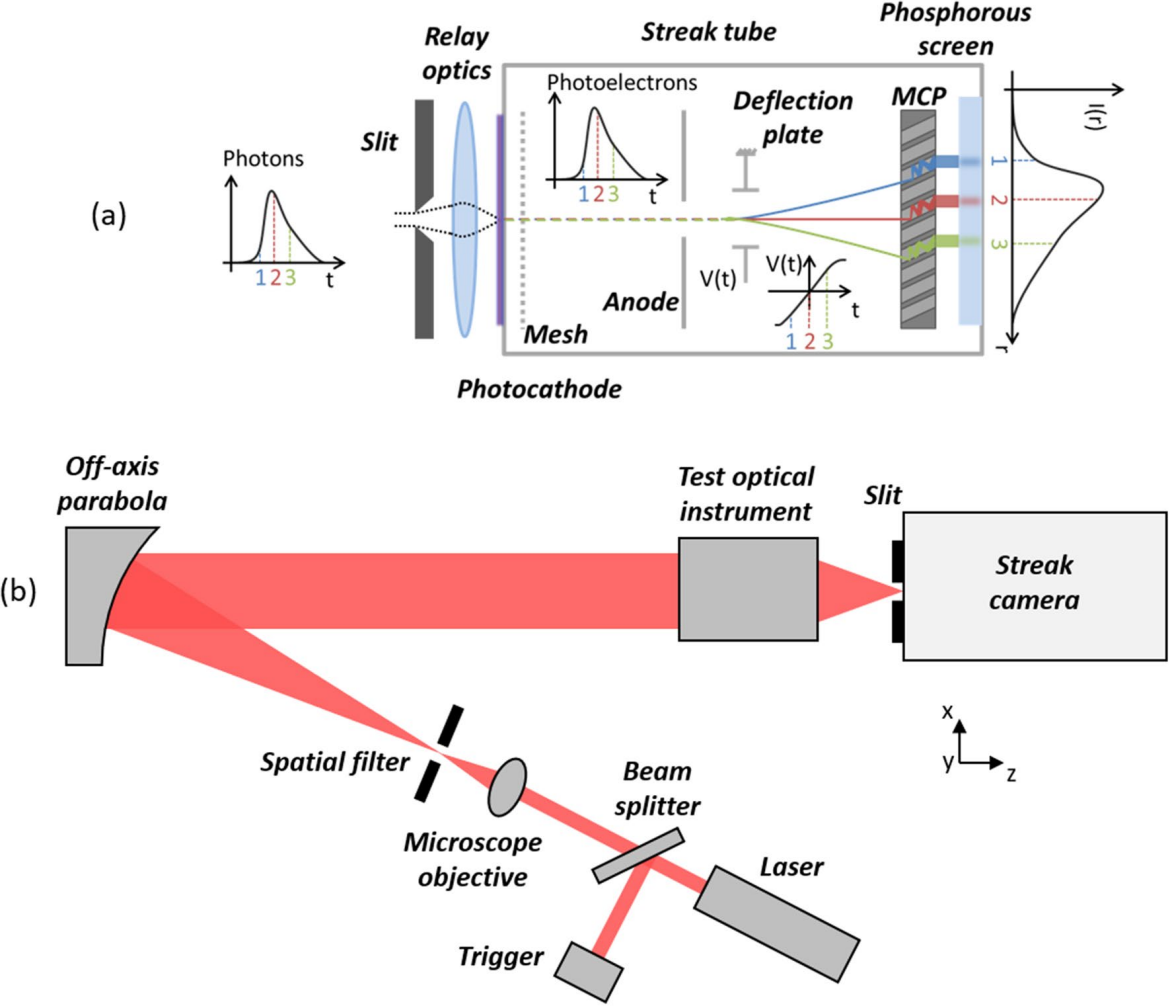
(**a**) Principle of a streak camera. Light at the slit is imaged on a photocathode with a relay optics. It creates photoelectrons which are deflected differently as a function of their time of arrival so that the temporal behavior of photons is measured. (**b**) Simplified sketch of the experimental setup. A femto-second laser beam is expanded to illuminate the optical instrument under test. At its focal plane, a streak camera records the temporal evolution of light along a slit.

The experimental setup is shown in Fig. 2b. A fs-pulsed laser beam goes through a beam expander to form a large collimated beam, illuminating the optical instrument to characterize. Figure 1 shows the sketch of the optical instrument under test: a typical zoom configuration followed by an optical window, mechanically supported by spacers inside a tube. The different optical surfaces are labelled *a* to *f*. The instrument is illuminated at a small field angle along *x* (0.73°), producing a non-rotationally symmetrical stray light profile at the detector. A streak camera (Fig. 2a) is placed with its slit oriented along *y* at the focal plane of the instrument. At its output, the camera displays an image with a temporal and spatial dimension. At each position *y*, we obtain a temporally stretch signal *I*(*t,y*). With a typical 100 fs laser pulse duration, a temporal resolution of a few ps is reached.

**Figure 3:**
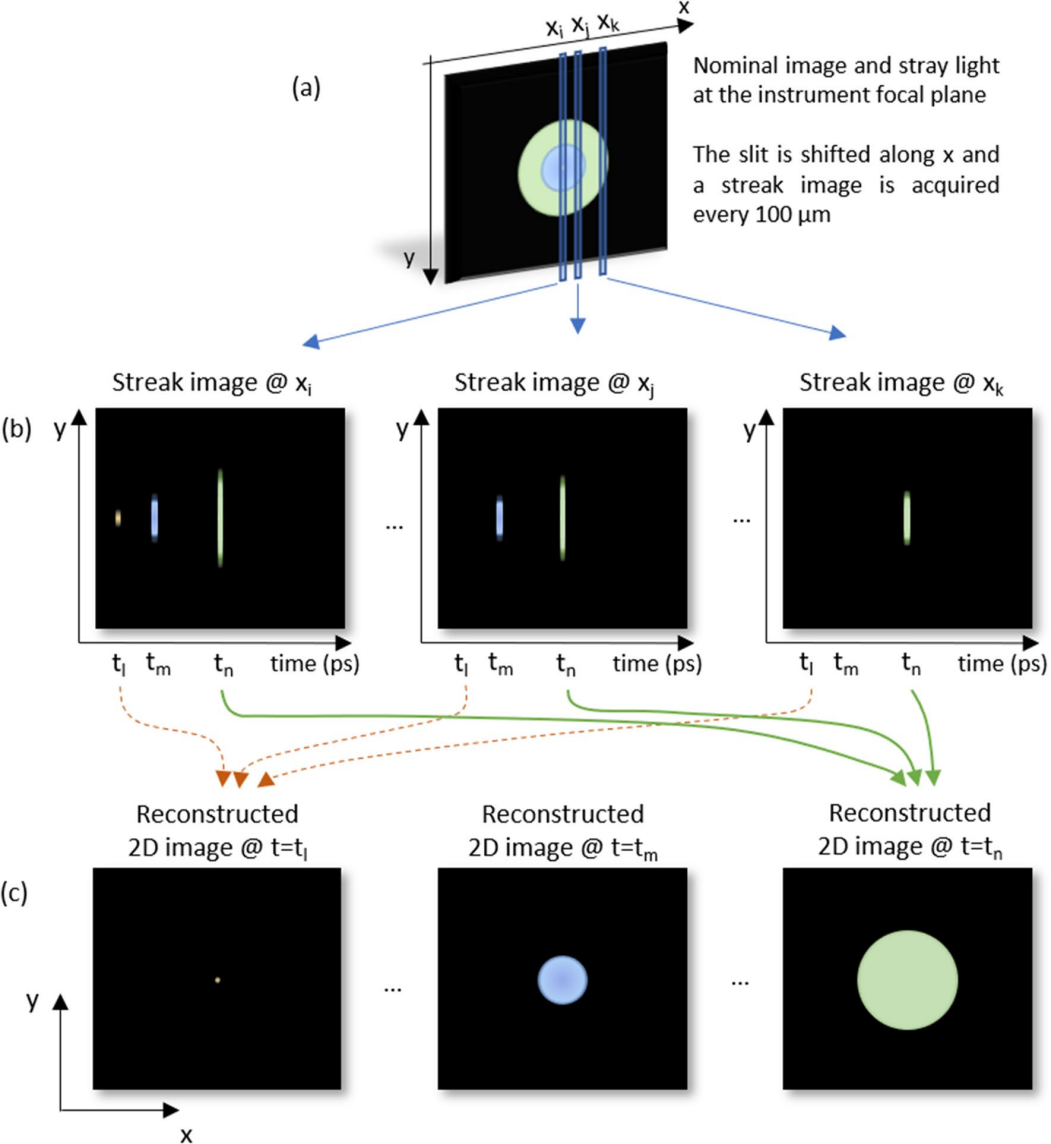
SL acquisition and reconstruction principle. Along a slit, the streak camera acquires the temporal behavior with a picosecond temporal resolution. The slit position is shifted along *x* and a temporally resolved streak image is acquired every 100 µm. From there, a movie of the SL reaching the detector is reconstructed.

The principle of the temporal SL characterization is shown in Fig. 3. The slit of the streak camera is scanned in the focal plane along *x* (a), acquiring streak images *I*(*t,y*) with steps *dx* of 100 µm (b). The different images are then recombined into a temporal sequence of 2D maps I(*x*,*y*) (c). A movie of the SL reaching the detector is obtained. The map I*(x,y)* at time *t*_*l*_ shows the nominal image (focused spot for an object at infinity) while at time *t*_*m*_ and *t*_*n*_ we get images of different SL contributors.

### Theoretical SL map

**Figure 4:**
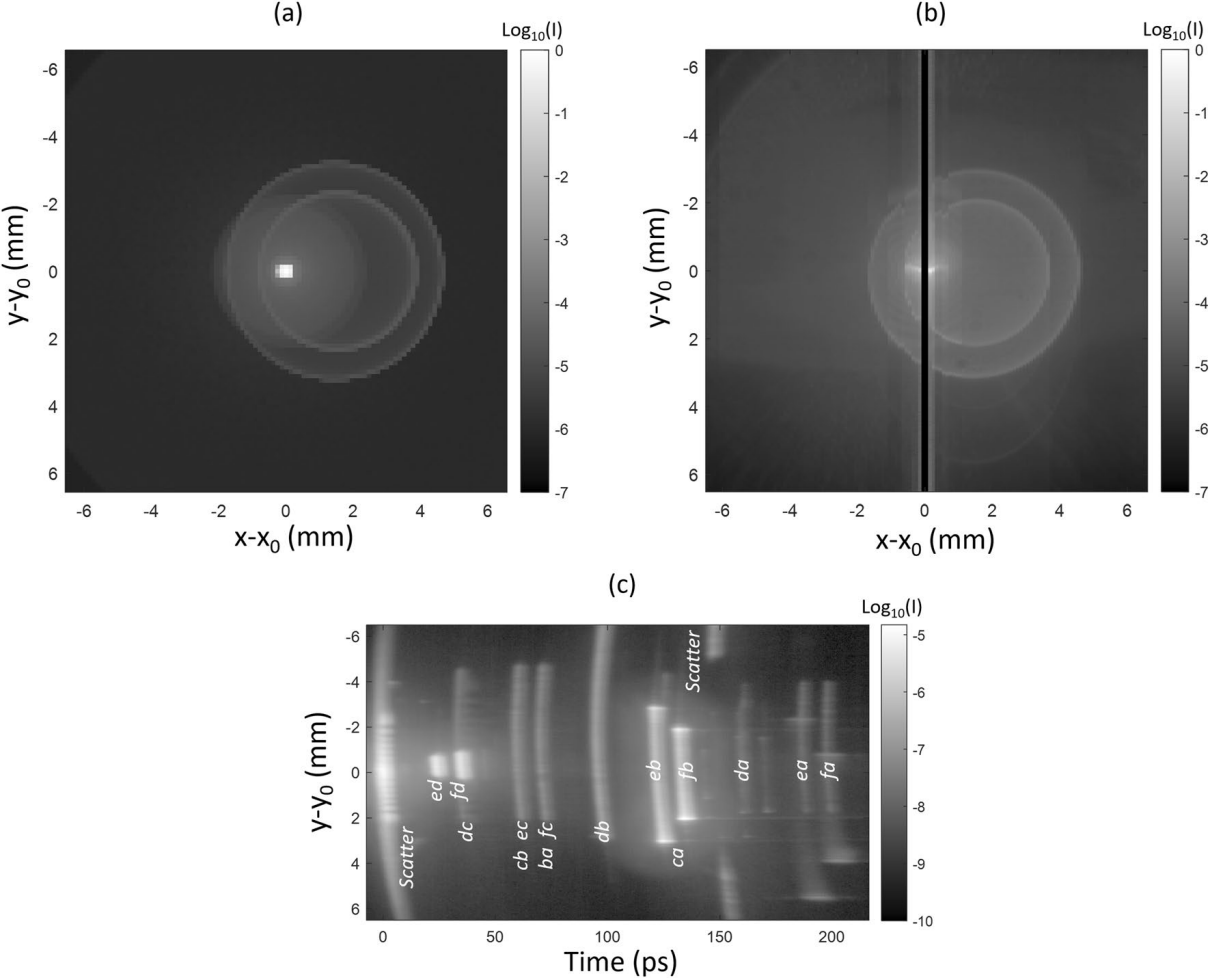
Theoretical (**a**) and experimental (**b**) irradiance maps (*I*), when the instrument under test is illuminated with a collimated beam with field 0.73° along *x*. The theoretical result is obtained by ray tracing, considering second level ghosts and scattering by the lens roughness. The experimental result is obtained by integrating the SL movie over the time domain. (**c**) Experimental streak image acquired with the slit at a distance 700 µm from *x*_*0*_.

In order to understand our results, Fig. 4a shows the theoretical irradiance map at the focal plane, as it would be measured with a regular 2D detector. It presents a bright spot at the position of the nominal image (*x*_*0*_,*y*_*0*_), surrounded by several SL ghosts decentered along *x* (because of the 0.73° field angle) and by others widespread over the focal plane.

### Streak image

Figure 4c shows a streak image *I(t,y)* with the slit at 700 µm from *x*_*0*_. It presents several features at different times, with different irradiances and sizes. The features present a curvature if all the rays in their underlying SL path do not reach the focal plane simultaneously. While the nominal spot arrives at time *t* = 0 ps, the streak image is acquired with the slit slightly beside. Indeed, the dynamic range of the camera is insufficient to display simultaneously the nominal spot and SL. Close to 0 ps, scattering due to surface roughness of the lenses creates SL over the entire slit, with an irradiance decreasing away from *y*_*0*_. The most significant ghost paths usually involve only two partial reflections (second level ghosts). In on-axis systems, there are *α = N(N − 1)/2* paths of that sort^1^, where *N* is the number of refractive interfaces. Here, *α* = 15 but only 14 of them are easily identified by the OPL on the streak image. Mapping of the features to their origin is done by comparing their time of arrival with the theoretical OPL of each SL path, predicted by ray tracing. The missing path is the ghost *ef*, occurring inside the optical window. The latter creates a slightly defocused ghost around the nominal image, which is not measured as the slit is too far from *x*_*0*_. At around 150 ps, a ring is present (no light for *y* values close to *y*_*0*_) and corresponds to scattering on the housing. The streak image can also present much weaker features from paths involving more than two partial reflections (for instance at 170 ps, between *da* and *ea*). In our setup, such higher-level ghosts can be generated either by the instrument under test or by the streak camera relay optics itself. Usually, these are negligible compared to second level ghosts.

### SL movie

**Figure 5:**
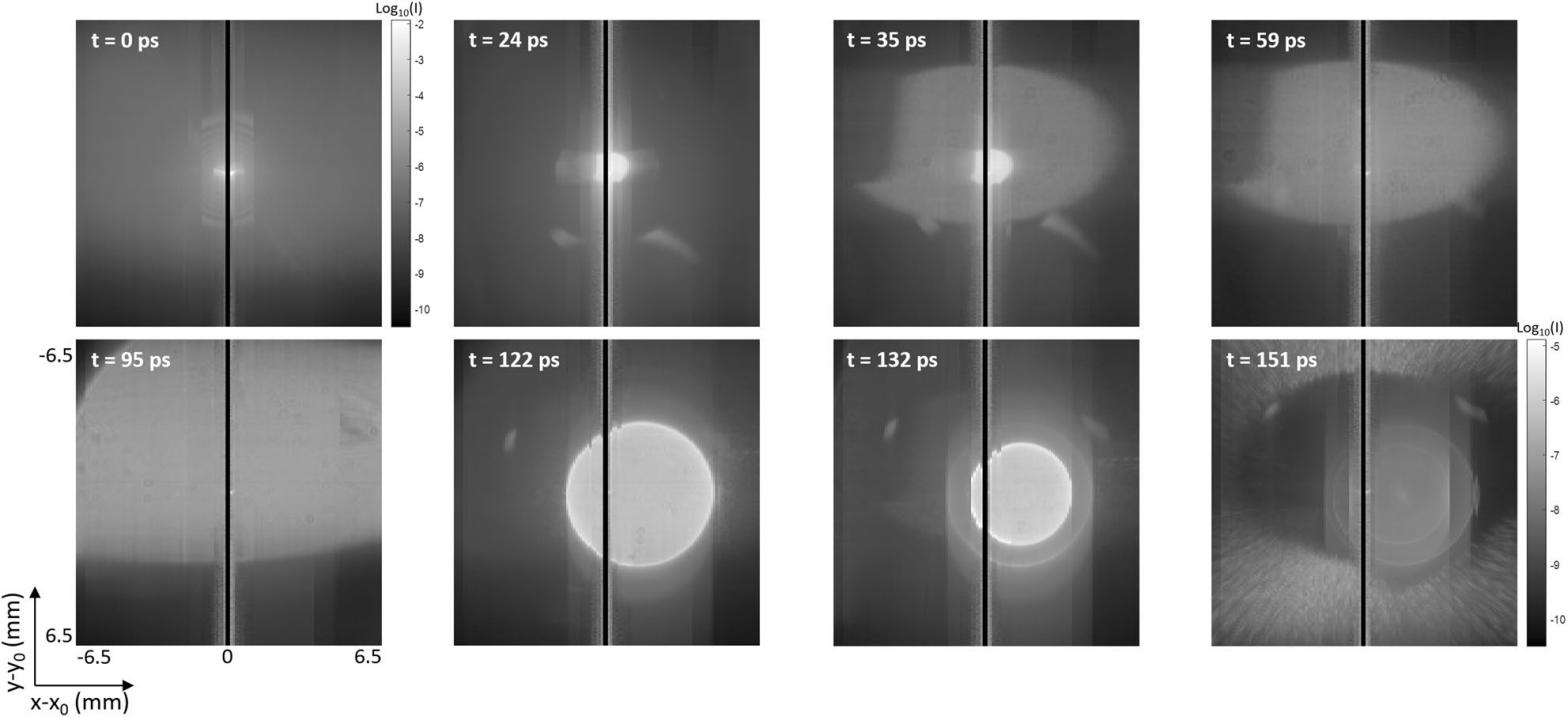
Screenshots of the SL movie at different times: scattering by lens roughness (*t* = 0 ps), ghosts (*t* = 24 to 132 ps) and scattering on the housing (*t* = 151 ps).

The full SL movie reconstituted by the streak images is available as supplementary material. In Fig. 5, we show screenshots *I*(*x*,*y*) of the SL movie at different specific times. A black vertical stripe is present at the center because the streak image *I(y,t)* is not measured at *x*_*0*_, as explained above. The first (upper left) figure of the series is at *t* = 0 ps. It shows an intense light around the image center at (*x*_*0*_,*y*_*0*_) as well as a quite uniform irradiance pattern all around. This is the result of a first SL component attributed to scattering due to lens roughness. This SL does not experience any delay due to secondary reflections. The other figures, obtained between *t* = 24 ps and 132 ps, show the decomposition of various ghosts. In some cases, different ghosts have similar optical path lengths and reach the detector simultaneously. It is the case at *t* = 35 ps where *dc* and *fd* are superposed while easily distinguishable (the latter is smaller and brighter than the former). On the contrary, at *t* = 59 ps, ghosts *cb* and *ec* are superposed and indistinguishable. Another remarkable pattern is the one shown at *t* = 151 ps: a large external ring is visible and is likely to arise from the scattering by the optical instrument housing. Summing the images *I*(*x*,*y*) for all times *t*, we obtain the total irradiance at focal plane as shown in Fig. 4b which is comparable to the theoretical SL map in Fig. 4a.

### Characterization

**Figure 6:**
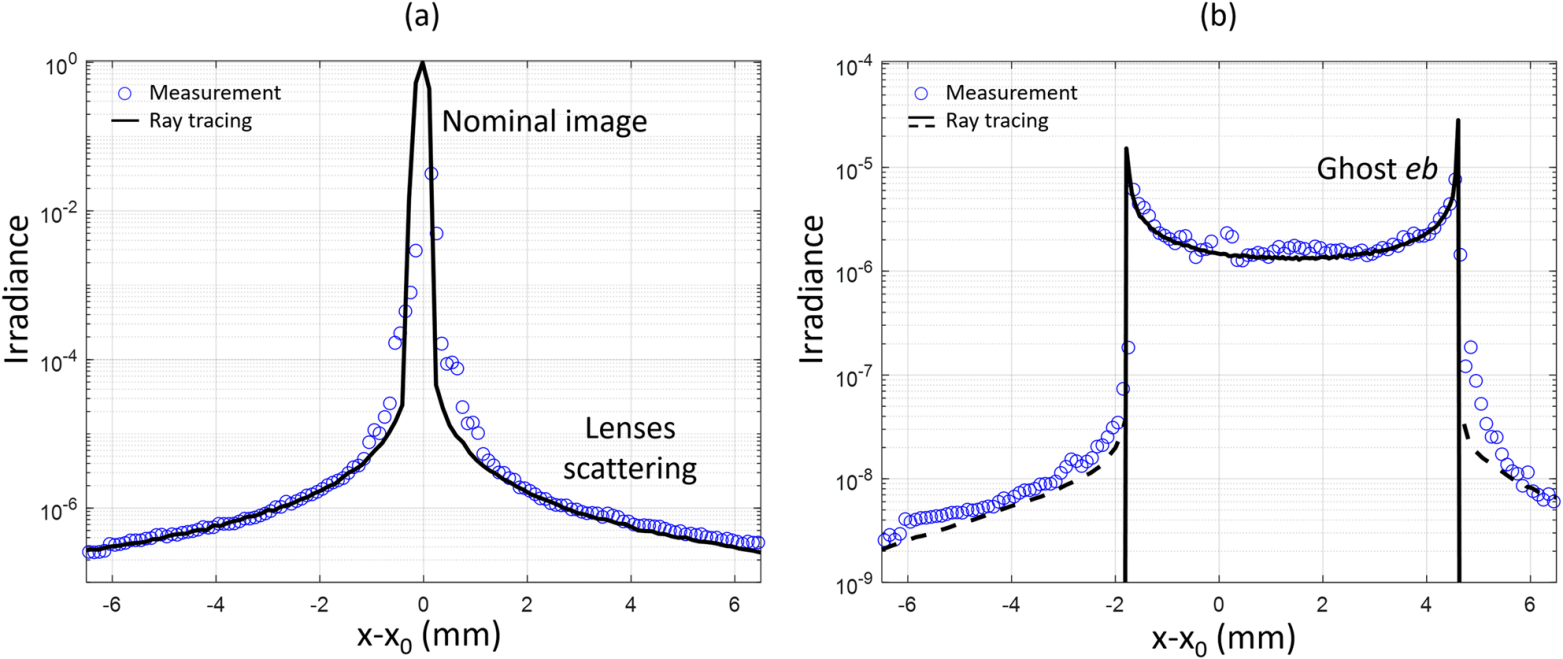
(**a**) Profile along *x* of the nominal image and scattering by lens roughness. The experimental results are compared to ray tracing results, obtained by considering a Harvey model BSDF with a 2.04 nm effective roughness. (**b**) Profile along *x* of the ghost *eb*. The experimental result is compared to the ray tracing result obtained by considering two specular reflections (continuous line) or considering also the scattering of ghost rays (dotted line).

The last step of our work was to consider the use of this measurement method to understand the origin of SL (ghost or scattering). Furthermore, we meant to determine whether these observations match with ray tracing simulations and scattering modeling, as a prelude to reverse engineering the SL origin. Figure 6a shows the profile of SL along *x* due to scattering on the lens roughness at *t* = 0 ps. The fact that the scattered light reaches the focal plane at a time which is function of *x*, as shown by the curvature of the scatter feature in Fig. 4c, was taken into account. The profile contains the SL from lens roughness, as well as the nominal image as they both arrive at *t* = 0 ps in (*x*_*0*_,*y*_*0*_). Reverse engineering was performed to estimate the bidirectional scattering distribution function (BSDF) of the lenses, initially unknown. For that, we start with an initial guess for the BSDF and compute the corresponding SL profile at the focal plane. Then, we iteratively adapt the model until it fits the experiment. A Harvey model BSDF^1,31^ with 2.04 nm effective roughness for each lens interface yields ray tracing results similar to the experiment.

Figure 6b shows the profile along *x* for ghost *eb* (t = 122 ps). The continuous black line shows the theoretical profile, obtained by ray tracing the specular reflections at lens interfaces. With uncoated lenses, the reflectivity is modeled by Fresnel equations, for which the results are in excellent agreement. In addition, the experimental profile presents a signal around the ghost. Usually neglected in simulations, this comes from the fact that ghost rays are partly scattered. Performing the ray-tracing with the BSDF model derived above, this behavior is reproduced, as shown by the black dotted lines.

## Discussion

Ultrafast ToF SL characterization is demonstrated in the case of an imaging instrument. We were able to isolate and identify ghosts, scattering from lens roughness and scattering from the housing. Hence, individual SL contributors can be compared with the theoretical model. Reverse engineering can be performed to retrieve information on optical properties, as it was carried out in this study with the scattering from lens roughness. With raw lenses, partial reflections are modeled with Fresnel equations and ghosts are therefore predicted accurately. With coated lenses, the comparison between measurement and theory could serve as a means to verify coating performances. Similarly, for SL due to scattering on non-optical surfaces, comparison between experiment and theory could emphasize an underperforming black surface treatment. Practically, this tells which surface and associated optical properties should be improved to reduce the SL to the expected level.

For optical instrument characterization, this method significantly improves the understanding of the SL. It is not only the total SL that can be measured and compared to performance requirement. In fact, because the different contributors are accessed separately, they are identified and can be compared to their respective expected level. Afterward, the SL model can be adapted accordingly. In the case where the total SL is larger than expected, this allows to identify the problematic contributor and to act upon it. When an unexpected feature is present, the optical path length is used to reverse engineer its origin. While this is not a trivial task to perform, the OPL is a useful clue to determine the surfaces involved in a SL path. For example, a feature with a small OPL necessarily involves surfaces close to each other, and reversely. Then, once the origin is found, classical SL control methods can be applied, for example by adding a baffle. Finally, it can happen that some of the detected SL comes from the measurement facility (e.g., ghost in a lens at the source, or scattering on the walls around the facility). If a feature is identified as such, it can be eliminated from the measurement by removing the signal at the corresponding time. For example, if a SL path with an OPL significantly larger than any dimension in the optical system under test is detected, it is likely that it is an external SL path.

Our method is applicable to a broad range of situations. In off-axis instruments, straight shots can occur when direct light reaches the detector without following the sequence of elements intended by the design. For instance, in a multi-mirrors system, this occurs when light is reflected on the first mirror directly toward the detector. In that case the SL path has an OPL shorter than the nominal image, therefore arriving sooner at the detector. In the case of instruments with diffractive elements, this method could also identify SL coming from unwanted diffraction orders. In each case, the knowledge of the origin is used as a baseline to determine the actions to undertake.

In this paper, the method is applied to a relatively simple optical system. However, it can be generalized to more complex systems as well. In the case of instruments with a larger number of optical elements, ToF measurements could emphasize a larger number of peaks as the number of possible SL paths is increased too. This can lead to ambiguities if multiple SL paths have an identical OPL, hence advanced mathematical tools will need to be developed. Nevertheless, SL features with the same OPL could be distinguished spatially if they are located on different areas of the focal plane. Finally, recent advances in optical design (e.g., freeform optics^32^) allows to build high performing compact instruments with a reduced number of optical elements. In that case, the ToF measurement is not necessarily harder to interpret, regardless of the complexity of individual optical elements.

Ultrafast ToF SL characterization is necessarily performed with monochromatic light, as a pulsed laser must be used. Nevertheless, streak cameras are available from the X-ray to the near-infrared domains and tunable pulsed lasers are available in an increasing wavelength range. Therefore, SL characterization is not limited to a single wavelength. Finally, instrument size can vary widely, with SL components whose OPL is comprised between a few millimeters and several tens of centimeters. In the case of instruments with very large OPL (e.g., for example large reflective telescopes), smaller temporal resolution can be sufficient, opening the possibility to use other sensors such as SPADs^8^ with high spatial resolution.

Besides SL characterization of optical instruments, another application of this method concerns the validation and improvement of traditional SL measurement facilities. When an instrument is to be tested, it requires a facility with very low SL. Otherwise, the SL measurement may contain contributions from the facility itself which cannot be distinguished with traditional methods. This happens frequently, in particular because SL facilities are usually validated by simulations only. Hence, this method would be particularly useful as it would allow for characterization of the SL in a facility and derivation of its origin, therefore contributing to improving it. Practically this could be envisaged by replacing the source of the facility by a pulsed laser and by placing the ultrafast sensor in the way of the output beam.

To conclude, ToF imaging with high temporal resolution offers new possibilities for SL characterization, in-line with the trend of high-end instruments requiring better SL control. By enabling decomposition and identification of SL components with the OPL, it breaks the status quo of conventional methods whose purpose is only verification. It solves a decades-old problem and offers the ultimate characterization and understanding of SL properties in optical instruments.

## Methods

### Laser source

The source is a Titanium Sapphire 780 nm pulsed laser with a 400 mW optical power (Tsunami from Spectra-Physics). It has a repetition rate of 81.2 MHz and a pulse duration of 100 fs.

### Streak camera

The streak camera is the *Optoscope SC-10* from the company Optronis GmbH, set in synchroscan mode. The streak tube is equipped with an S25 photocathode with quantum efficiency of 12% at a wavelength of 780 nm. The slit is adjusted with a width of 60 µm along *x*, and a length of 13 mm along *y*. The sampling along the slit is of 14.47 µm and the temporal behavior is measured with a sampling of 210 fs per pixel.

### Test instrument

The optical instrument under test Fig. 1 consists of a biconvex lens (surface radii of curvature *R1* = 24.02 mm and *R2* = − 134.6 mm; thickness *e* = 6.5 mm), followed by a plano convex lens (*R1* = − 38.6 mm; *R2* = ∞; *e* = 3.5 mm) and an optical window (*e* = 1 mm). The different elements are made of N-BK7, uncoated and with a clear aperture of 25.4 mm. They are mechanically supported by spacers inside a lens tube of respectively 8 mm and 4 mm. The optical combination has an effective focal length of 63.3 mm and a back focal length of 33.2 mm.

### Acquisition and processing

The collimator has a diameter of 100 mm and is placed at 1000 mm from the optical instrument under test. The scan along the *x*-direction is performed by translating the tested instrument while the camera and the slit remain in place. The reason is that the instrument is much lighter than the camera. To avoid vignetting of the SL features, the input beam must have a sufficiently large diameter so that the instrument can be uniformly illuminated by the beam. Optical densities are placed in the way of the beam to adjust the input power for the signal to be below saturation of the streak camera. This enables the measurement of SL over a large dynamic range. The different acquisitions performed during the scan are then recombined into a 2D image.

### Photon efficiency

In the experiments presented here, we were not limited by photon-efficiency and we demonstrated measurements of SL features over a dynamic of 10^9^. Indeed, in our experimental conditions (i.e., mean power of 400 mW with integration time of 10 s), the total number of photons sent is in the range of 1.6 × 10^19^. Consequently, the maximum achievable dynamic can be very high. For example, a feature at dynamic of 10^15^ would mean that about 1000 photons are measured at every pixel, considering the quantum efficiency. This is much higher than the noise of a typical S25 photocathode, which is of the order of a few tens of photons per cm^2^∙s in photon counting mode.

### Scattering modeling

Lens roughness BSDF is fitted with a Harvey model^1,31^ whose profile is given by Eq. (1). It depends on the scatter angle $\theta_{s}$, the incident angle $\theta_{0}$ and the three parameters *b*, *s* and *L*. Equation (2) gives the associated total integrated scattering (TIS) and effective roughness $\sigma_{eff}$. Here, the fit gives *b* = 55.395, *s* = - 1.55 and *L* = 0.00078, corresponding to an effective roughness of 2.04 nm.

1 $$BSDF\left( {\theta_{s} ,\theta_{0} } \right) = b \cdot \left[ {1 + \left( {\frac{{\left| {\sin \theta_{s} - \sin \theta_{0} } \right|}}{L}} \right)^{2} } \right]^{s/2}$$

2 $$TIS = \frac{2\pi b}{{L^{s} \left( {s + 2} \right)}} \cdot \left[ {\left( {1 + L^{2} } \right)^{{\frac{s + 2}{2}}} - \left( {L^{2} } \right)^{{\frac{s + 2}{2}}} } \right] = \left( {\frac{{4\pi \sigma_{eff} }}{\lambda }} \right)^{2} .$$

## Supplementary Information

Supplementary Video 1.

Supplementary Information 1.
