# Supplementary material for: Stray light characterization with ultrafast time-of-flight imaging
Source: Sci Rep. 2021 May 12;11:10081. doi: 10.1038/s41598-021-89324-y (PMC8115156; doi:10.1038/s41598-021-89324-y)
Supplement: Supplementary file 2 — Supplementary Information 1. [file 41598_2021_89324_MOESM2_ESM.docx]

**Legend for the supplementary material.**

The video in supplementary materials is a SL movie. It shows the experimental SPST map as a function of the time, where ghosts and scattering contributions appear successively as they arrive at the instrument focal plane. The screenshots presented at Fig 5 are extracted from this movie.
